# Morphine and Scopolamin as a Preliminary to Anesthesia

**Published:** 1906-03

**Authors:** K. Lowder Reid

**Affiliations:** Atlanta, Ga.


### MORPHINE AND SCOPOLAMIN AS A PRELIMINARY TO ANESTHESIA.*

### REPORT OF SIXTY CASES OF ETHER AND FIVE CHLOROFORM ANESTHESIA, PRECEDED BY MORPHINE AND SCOPOLAMIN.

BY K. LOWDER REID, M.D.,

ATLANTA, GA.

I was led to try morphine and scopolamin as a preliminary to anesthesia by reading a report of sixty-five cases, by Selig of St. Louis, last summer.

I have not picked the cases in which to use morphine and scopolamin, but have taken them as they came. About half of these have been abdominal cases. There have been several long anes-

*Read before the Fulton County Medical Society.

thesias, and several serious cases with patients in bad condition to start with. I have used from $\frac{1}{8}$ to $\frac{1}{4}$ gr. morphine, and from $\frac{1}{200}$ to $\frac{1}{100}$ gr. scopolamin, in most cases $\frac{1}{6}$ gr. morphine and $\frac{1}{100}$ gr. scopolamin.

It is given hypodermically about half an hour before the commencement of anesthesia, a longer period than half an hour being preferable to a shorter, as it takes as much as half an hour to get the full effect of the drugs.

In most of the ether cases the nitrous oxide gas was used as a preliminary, but in several ethyl chloride was used, instead of nitrous oxide.

The patient is usually in a better frame of mind after taking morphine and scopolamin, quieter and less nervous, and consequently he goes under the anesthetic more quietly and rapidly.

Decidedly less of the anesthetic is necessary in cases that have had morphine and scopolamin, and a lighter anesthesia can be satisfactorily maintained, as they are not so apt to vomit and resist when only lightly anesthetized. The anesthesia is usually quieter and more cases approach the ideal condition, one resembling normal deep sleep.

There is much less tendency to the secretion of mucus by the air-passages; a very decided advantage. Nausea and vomiting occur less frequently than without morphine and scopolamin, the retching and vomiting that so often occur when the patient is being moved from the operating table have been absent in every or almost every case in which morphine and scopolamin has been given, and very few cases have been nauseated when they waked. Usually the patient sleeps for an hour or two after the operation, often for several hours, and awakes quietly and comfortably, and without mental disturbance or distress.

In some cases this combination, morphine and scopolamin, abolishes the corneal and pupillary reflexes before surgical anesthesia is reached, so that other signs of anesthesia must be watched in order to judge the depth of anesthesia.

Of course these drugs have not yet been used in this way in a sufficient number of cases to establish their value with absolute certainty, but they are well worth trial, and so far I have seen no bad results from them, but have been very much pleased with their effect.

DISCUSSION OF DR. REID'S PAPER.

*Dr. Goldsmith:* I have had only a limited experience in the use of morphine and scopolamin as a preliminary to anesthesia, but I feel satisfied that the good comes from the morphine.

In two cases where it was given for me, it did not have as good effect as the morphine alone, as there was some nerve disturbance following its administration. I give gr. $\frac{1}{6}$ or gr. $\frac{1}{4}$ of morphine with or without the atropine just before laparotomy with the most happy result. The patient approaches the operating-table in quieter manner and with more fortitude.

I should like to know if scopolamin has ever been used without the morphia as a preliminary to anesthesia?

*Dr. W. E. Campbell:* I have not used scopolamin in the manner spoken of in the paper read by Dr. Reid, but have used it in my eye work where I have been in habit of using atropin. It is an excellent mydriatic but on account of its toxic effect I prefer to use the atropin, which is, I consider, a much more satisfactory drug.

*Dr. E. C. Davis:* My experience has been limited in the use of these drugs in the manner spoken of, but I will say that I am certain that the scopolamin does away, to large extent, with the collecting mucus we have to contend with in operations. I consider the use of morphine or morphine and atropin prior to a laparotomy a dangerous procedure as a rule. I do not like to use anything that interferes with peristalsis, but I am not as opposed to this as formally. I prefer the use of codeine for pain after operation and have used morphine at this stage.

*Dr. M. A. Purse:* For years I gave anesthetics for the late Dr. V. O. Hardon. It had been suggested to me the use of morphine and atropine, prior to anesthesia, by the elder Dr. J. McF. Gaston, who is now also dead. I used it in a series of twenty-five cases with happy effect. But this combination will not relieve the nausea after operation and if scopolamin will do what morphine and atropine will not do, so far as relieving nausea, I don't think the reader of this paper should hide his light under a bushel.

*Dr. W. A. Selman:* I have had no experience with scopolamin but have used hyoscine with good effect—my patients took very much less ether.

*Dr. W. S. Goldsmith:* My question has not been answered yet as to whether scopolamin had ever been used without morphine. Like Dr. Davis, I too considered at one time that morphine before any abdominal operation was dangerous, but I have modified my views very much on this subject, and now use it very often.

*Dr. K. L. Reid* (in closing): In answer to Dr. Goldsmith's question, will say, I have not used scopolamin by itself. Will also state that I have used morphine before abdominal operations in twenty-five or thirty cases and have had no trouble with the bowels whatever after the operation.

